# ASSESSMENT OF THE AGEC STANDARD DEMONSTRATING STUDENT LEARNING

**DOI:** 10.1093/geroni/igz038.1472

**Published:** 2019-11-08

**Authors:** Robert J Maiden, Jan Abushakrah

**Affiliations:** 1 Alfred University, Alfred, New York, United States; 2 Portland Community College, Portland, Oregon, United States

## Abstract

Addressing the gerontology program’s experience in measuring and integrating the competencies in their curricula is a fundamental challenge in program evaluation. Using the AGHE Gerontology Competencies for Undergraduate and Graduate Education is the key. We will demonstrate how one identifies the learning outcome measures across the curriculum based on the competencies by adumbrating a four-step process. First, it entails developing a written statement of the key learning outcomes, expressing them in objective, measurable terms. Second, the learning outcomes are assessed. Third, the results of the assessments are posted in a matrix format across a four or five year period. Fourth, the results of the learning outcome assessments are discussed, evaluated, and implemented in a formative process to improve teaching and learning. In addition, the results can be applied in a summative way to evaluate and improve the gerontology program.

